# In the Surgery at Guy's

**Published:** 1889-06-01

**Authors:** 


### The Hospital Workshop.

### No. II.—IN THE "SURGERY" AT GUY'S.

(By our Special Commissioner.)

Near the entrance of most general hospitals is a room where patients are received—the lesser cases being attended to at once, while the more serious are sent on to the wards. At Guy's this room is called the "surgery," and the work—which consists chiefly in the treatment of accidents—is carried out by students, under the direction of a junior house surgeon. For the most part the patients are drawn from the working classes, and many come from the wharves, the dockyards, and the factories, with which the district abounds.

During the course of a morning visit, a short while back, much interesting information was afforded to your commissioner by the courteous house surgeon, Mr. G. B. Smith, in charge of the "surgery." A few hours spent in that busy place will teach more about the lives of the toilers than could be learned from a month's study of books or of newspaper articles. Moreover, the immense amount of good that is done by institutions of this kind cannot fail to be brought home to the sympathetic observer.

The object of our visit had hardly been explained, when a fair-haired, good-looking young Yorkshireman presented himself with his hand tied up in a handkerchief. He told us calmly enough that a revolver had been brought for repair to the shop where he worked. While examining the pistol, which was said to be unloaded, our patient let fall the hammer, and exploded a cartridge. The bullet struck the front of his left middle finger, and appears to have glanced off the bone, leaving a ragged blackened wound, which requires no treatment beyond a simple antiseptic dressing. Injuries like this usually take a long time healing, and are apt to leave stiff fingers. The case points the old saw, "familiarity breeds contempt." For how else could a young man who has spent seven years in a gunmaker's shop be ready at the end of that time to take a stranger's word that a weapon, handed casually over the counter, is not loaded? However, the mischief has been done, and luckily without any very serious injury. Considering the general disuse of weapons that characterises modern times, it is most remarkable the number of gunshot wounds—often with very queer or dubious histories—that are brought to the London hospitals. A well-known surgeon tells of a patient who accounted for a furrowed wound along the scalp by saying his "pal" had "fired a trifle low." The man earned his living in an East End booth by having objects shot off his head after the method brought into vogue centuries ago by William Tell.

At Guy's, besides the new cases that drop in any time of the day or night, those requiring further treatment come up every morning at ten o'clock. Here is one of them. A faded young woman, evidently very poor, who carries a small child in her arms. It seems that three days ago her little girl "fell on a quart can, and the wire on top cut her lip open." She was brought here, and the "doctors" put in a stitch, but the child has managed to pull it out during the night. The lower lip now shows a gaping wound, which the dresser deftly wires together and seals up with a bit of lint dipped in collodion. This little operation is soon over, and cannot be very painful, but still it caused a great outcry for a few minutes. At length a rough-looking man, with two black eyes and a bandage round his head, jumped up abruptly, and, pulling an orange out of his pocket, gave it to the child. In spite of tears and woe, baby clutched the prize firmly, and a little later handed it to her mother to take care of. Children's troubles are like April showers, and, with just an atom of prompting, she waves a "ta, ta" to the kindly house surgeon.

A chair is placed in the centre of the room, where patients are seated for dressing or for examination. It is rarely vacant for any length of time during the day, and mother and child have scarcely reached the door before their place is taken by a great brawny engineer, who holds out his hand, showing a cut that reaches from fingers to wrist. He tells us that "while putting a strap upon a machine a nail on the strap was pulled right through his hand." There is no time to waste in the "surgery," so a dresser at once syringes out the wound with an antiseptic lotion, and brings the edges together with half-a-dozen stitches of silver wire. This proceeding the patient watched like a stoic, but when the dresser spoke of the need of one more suture volunteered that "he did not think the place deep enough to want another." That remark was the only suggestion of all the pain he must have undergone, and he made it without a quiver of the voice, almost, indeed, as if the hand belonged to someone else, in whom he took a friendly interest.

In this class of practice, speaking broadly, results are not altogether satisfactory. Many of the patients are dissipated and unhealthy, their wounds are often poisoned, and they are careless of after treatment, going back to work too soon or neglecting the proper dressing. Of late years a vast improvement has no doubt been effected by the use of antiseptics, but still the surgeon who has to treat the poorer town-dwellers is heavily handicapped by the quality of his material. Yonder comes a young man limping in slowly and painfully, with a face that shows unmistakable traces of drink and hard living. While at work on a new building close by, an iron girder has fallen on his foot and crushed the nail off the big toe. He is not in a sick or benefit club, so that he will draw no money for the fortnight during which he is likely to be laid up. The idea of a claim for compensation does not seem to have occurred to him, or else he knows too well the hopelessness of proceeding under the present Employers' Liability Act.

The name of each patient, with the nature of the case, is entered in a book, and a glance down its columns gives one a fair idea of the work done here day by day. There is a large proportion of "foreign bodies," and such entries as "lime in the eye," "pebble in nose," "bone in throat," are common. Burns, bruises, scalds, cuts, crushes, sprains, and broken bones follow each other without end. There is a fair number of "scalp wounds," especially on Saturday nights. Here is a note—"fractured olecranon," a rather infrequent break of the elbow; and there on the next page is a similar entry. This running together of rare cases is an extraordinary fact, known to all who have had much experience of hospital practice. Some striking cases were mentioned by the house surgeon. For years he had not seen a fractured shoulder-blade, and then within a single month four patients came to Guy's suffering from that injury. But the crowning instance was the appearance of three dislocated elbows on three consecutive days. Facts of this kind are beyond question, and would afford the curious a pretty problem in the doctrine of averages. It has been suggested that the medical man having seen a remarkable case is naturally on the look out for others of the same sort. That observation is sound, so far as it goes, but would not apply to such well-marked accidents as those mentioned, which could hardly escape notice under any examination, however careless.

But here is someone waiting to be seen. A girl of seventeen—pale, undersized, dejected—a typical weed of town growth. It is painfully evident that her nerves are highly strung, for while she is telling how she fell in the workshop and sprained her wrist, the sudden sneeze of a patient in the next room made her jump nearly off her chair.

She is able to muster up a faint smile, however, when told there are no bones broken, and that a bandage and a night's rest will put her all right again. And then the doors are thrown open, and a man is brought in on a stretcher. A companion tells what has happened, with much beating about the bush and emphasis on unnecessary points. Summed up shortly, his tale is to the effect that an iron casting, "a street watering post," fell, first on his mate's shoulder, and then on his foot, which is now painful, and he cannot walk. There are no bones broken, a fact which has to be shouted repeatedly into the patient's ear, for he is deaf as the proverbial implement which has caused the injury. "He was brought up in a deaf and dumb school, he was," remarked his friend, as he helped him away after the injured member had been securely cased in with broad strips of plaster.

The cry is "still, they come." A big, burly "lumper," or dock labourer, is stripped, and waiting in the side room. His muscular frame would make a fine model for a sculptor's Hercules. While carrying a sack of wheat across a plank he has slipped and fallen six or seven feet into the hold of a ship, and complains of damage to his side and shin. The case is fortunately not a serious one. Some strips of plaster half way round the body, and a simple dressing to the leg, and he may be sent home. That same leg, he tells us, was broken twelve years ago by a fall from the ladder of a gangway. He has had a lot of other accidents. Some time ago he broke several ribs on the same side he has come about to-day. "Always an unlucky side, that, sir." And once, after a fall which injured his spine, he had to attend as an out-patient for eleven months. Notwithstanding the constant risk to which these dock labourers are exposed, very few of them join benefit-clubs, and this patient said, candidly enough, in reply to a question, "No, I do not belong to one; and I must own I am a great fool that I do not." Yet this man, by his own statement, can sometimes earn, when trade is brisk, as much as ten or twelve shillings a day. On the other hand, during two or three of the winter months no work is to be obtained.

The interest of this place is simply inexhaustible. Temperance reformers would be able to collect plenty of evidence, were such needed, as to the terrible effects of drink amongst the poor. One nurse, an old inhabitant, went so far as to say that nearly all the women who came there were drunk; and added that sometimes a respectable man would follow his wife thither and ask despairingly, "What am I to do with a woman like this? She has such-and-such a number of children at home." Certainly, the disciples of Sir Wilfrid Lawson would find a wide field among the 14,000 cases treated here annually. Some inkling of the amount of work involved may be gathered from the fact that it costs the hospital over £300 a year for supplying the "surgery" alone with a particular kind of strong plaster, made of unbleached calico. A queer illustration of the warps and twists of human nature will be afforded to the cynic by the notice that occupies a prominent place, "Beware of Pickpockets." The crime of robbing a church dwarfs into insignificance by the side of filching in a casualty-room. Another notice intimates that patients are required to pay threepence at the time of receiving medical or surgical relief, and sixpence for a fortnightly attendance. The rule, however, seems to be more honoured in the breach than the observance, and the box which is placed to receive these contributions does not greatly swell the funds of the charity. Indeed, the plan seems unworthy of a richly endowed hospital like Guy's. Otherwise, one cannot express too much sympathy and goodwill towards the prompt, skilful, and kindly treatment accorded to these maimed and poverty-stricken sufferers.

